# Prognostic significance of CD45RO+ memory T cells in renal cell carcinoma

**DOI:** 10.1038/bjc.2011.368

**Published:** 2011-09-20

**Authors:** K Hotta, M Sho, K Fujimoto, K Shimada, I Yamato, S Anai, N Konishi, Y Hirao, K Nonomura, Y Nakajima

**Affiliations:** 1 Department of Surgery, Nara Medical University, 840 Shijo-cho, Kashihara, Nara 634-8522, Japan; 2 Department of Renal and Genitourinary Surgery, Hokkaido University Graduate School of Medicine, Kita 15, Nishi 7, Kita-ku, Sapporo 060-8638, Japan; 3 Department of Urology, Nara Medical University, 840 Shijo-cho, Kashihara, Nara 634-8522, Japan; 4 Department of Pathology, Nara Medical University, 840 Shijo-cho, Kashihara, Nara 634-8522, Japan

**Keywords:** renal cell carcinoma, memory T cell, CD45RO, tumour-infiltrating lymphocytes, prognosis

## Abstract

**Background:**

Memory T cells are well known to have a critical role for host defense in humans. However, their role in actual human cancer remains largely unknown. In this study, we tried to reveal the clinical importance of tumour-infiltrating CD45RO+ memory T cells in renal cell carcinoma (RCC).

**Methods:**

We analysed 105 patients with RCC, who received radical or partial nephrectomy. Those were 65 in TNM stage I, 7 in stage II, 15 in stage III, and 18 in stage IV, respectively. CD45RO expression was evaluated by immunohistochemistry. CD4 and CD8 expressions were also systematically assessed in the same manner.

**Results:**

Patients with higher TNM stage or high nuclear grade were found to have higher densities of CD45RO. Furthermore, CD45RO status was positively correlated with preoperative C-reactive protein level. In prognostic analysis, CD45RO+lo patients had a significantly better prognosis than CD45RO+hi patients. There was also a significant difference between CD4+lo and CD4+hi groups, whereas no significant difference was observed in CD8 T-cell status. Finally, multivariate analysis revealed that CD45RO+ status was the independent prognostic factor for patient overall survival.

**Conclusion:**

CD45RO+ memory T-cell status has a significant independent prognostic value, indicating that the adaptive immune response is functionally critical in human RCC.

Renal cell carcinoma (RCC) is the most common malignancy of the kidney and the incidence has been rising steadily. Due to the lack of symptoms at the early stages, about one third of patients present with advanced disease, either locally advanced or metastatic (Motzer *et al*, 1999; Cohen and McGovern, 2005). Although the early stage RCC is usually treated by surgery with good prognosis, the therapy for advanced-stage tumours still needs to be improved. Renal cell carcinoma is generally refractory to conventional anticancer treatments including chemotherapy and radiotherapy. It has been long expected that RCC is one of the feasible targets for immunotherapy. Although conventional immunotherapy such as interleukin-2 and interferon-*α* remains the effective therapy when treating advanced RCC, long-term clinical outcome has been disappointing (Motzer *et al*, 1999; Yang *et al*, 2003). Recent another immunotherapy trials including tumour vaccines and allogeneic stem cell transplants have shown a certain effect on the prognosis of patients with advanced RCC (Childs *et al*, 2000; Holtl *et al*, 2002; Avigan *et al*, 2004). New agents targeting vascular endothelial growth factor, platelet-derived growth factor, and mammalian target of rapamycin pathway have been demonstrated to have significant clinical response and prolong patient survival (Escudier *et al*, 2009; Motzer *et al*, 2009; Sun *et al*, 2010). Some of them have been also shown to be superior to cytokine immunotherapy such as interferon. Therefore, those targeted therapies currently represent the first-line standard for metastatic RCC. However, these treatments are not enough to achieve complete response and also have several adverse effects. Therefore, a deeper understanding of biology of RCC is required to develop more effective and less toxic systematic therapies for patients with advanced disease.

CD45 is known as the leukocyte common antigen and function as a tyrosine phosphatase in leukocyte signalling. The expression of different CD45 isoforms is cell type specific and depends on the stage of differentiation and state of activation of cells. In humans, CD45RA and CD45RO are thought to be naive and memory T cells, respectively (Akbar *et al*, 1988; Merkenschlager *et al*, 1988). Memory T cells are generated during cell-mediated immunity responses, and survive for many months and years after the antigen is eliminated. These memory T cells are responsible for more rapid and amplified responses to second and subsequent exposures to antigens. Memory T cells have critically important role in host defense for infection in humans. In tumour immunity, recent studies on colorectal and gastric cancer have demonstrated that high density of CD45RO+ tumour-infiltrating lymphocytes (TILs) is correlated with increased survival, and CD45RO+ TILs is independent prognostic factor (Pages *et al*, 2005, 2009; Galon *et al*, 2006; Lee *et al*, 2008). Although those studies have suggested that memory T cells have important role in tumour immunity, the clinical importance of memory T cells in RCC has not been previously addressed. Renal cell carcinoma has been considered to be an immunogenic cancer, with pathological specimens frequently large numbers of TILs (Webster *et al*, 2006). Several studies have previously reported that high densities of CD4+ or CD8+ T cells have worse impact on the prognosis in RCC, indicating functional importance of TILs (Nakano *et al*, 2001; Bromwich *et al*, 2003). However, the significance of each T-cell subset in RCC is still controversial, since results are not always consistent between different studies (Igarashi *et al*, 2002; Bromwich *et al*, 2003).

The aim of this study was to systematically investigate the importance of TILs using T-cell subset markers including CD45RO as well as CD4 and CD8, and to clarify the clinical significance of CD45RO+ memory T cells infiltrating in human RCC.

## Materials and methods

We analysed 105 patients with RCC, which received radical or partial nephrectomy during 2003 and 2008 at Nara Medical University Hospital. The age of patients ranged from 31 to 84 years (median 65 years) and the male to female ratio was 2.6 : 1.0. None of these patients received preoperative immunotherapy or renal arterial embolisation therapy. They were histopathologically composed of 84 (80%) of clear cell type, 9 (9%) of granular cell type, 5 (5%) of papillary cell type, 4 (4%) of spindle cell type, 2 (2%) of cystic cell type, and 1 (1%) of mixed clear and spindle type. The tumour stage was classified according to the UICC TNM classification of renal tumours (Guinan *et al*, 1997). Pathological grades were assigned according to the criteria proposed by Fuhrman *et al* (1982). We measured preoperative serum C-reactive protein (CRP) and defined patients with CRP of >0.5 mg dl^−1^ as positive group as previously described (Tatokoro *et al*, 2008; Saito *et al*, 2009). This study was approved by the Ethical Review Committee of Nara Medical University Hospital.

### Immunohistochemistry

Immunohistochemical staining for CD45RO, CD4, and CD8 was performed with a Dako Envision kit (DAKO Cytomation, Tokyo, Japan). Formalin-fixed, paraffin-embedded tissues were cut into 5 mm sections, deparaffinised, and rehydrated in a graded series of ethanols. Antigen retrieval was done by heating tissue sections using a Target Retrieval Solution, pH 9.0 (DAKO). Then, the samples were incubated for 5 min in peroxidase blocking solution (DAKO) to inhibit endogenous peroxidase. The sections were then incubated overnight at 4°C with anti-human CD45RO (UHL1, monoclonal mouse; DAKO), CD4 (1 : 40) (4B12, monoclonal mouse; DAKO), and CD8 (C8/144B, monoclonal mouse; DAKO). A subsequent reaction was carried out using secondary antibodies (DAKO) at 37°C for 30 min. Then, the sections were washed three times with phosphate-buffered saline and subsequently the colour was displayed with DAB (DAKO) for about 5 min. Sections were counterstained with haematoxylin, dehydrated in ethanol, cleared in xylene, and coverslipped.

### Evaluation of immunostaining

By immunohistochemistry, we evaluated tumour-infiltrating CD45RO, CD4, and CD8 T cells on RCC tissues as previously reported (Ohigashi *et al*, 2005; Anraku *et al*, 2008). We selected different five areas with most abundant positively stained cells in each tissue under 400 magnifications. Positive cells in the selected areas for each T-cell marker were counted independently by two investigators without knowledge of clinical information. In case of disagreement, the slides were re-examined and a consensus was reached by the investigators. Then, we calculated the median number of each sample.

### Statistical analysis

Comparisons among the clinical and pathological features were evaluated using *χ*^2^ and Fisher's exact tests. Statistical significance between two groups of parametric date was evaluated using an unpaired Student's *t*-test. Survival curves were estimated using the Kaplan–Meier method, and the significances of differences between survival curves were determined using log-rank test. Multivariate comparisons of survival distributions were made using Cox proportional hazards models. All tests were two-sided and *P*<0.05 were considered statistically significant.

## Results

### Density of TILs

**Figure 1:**
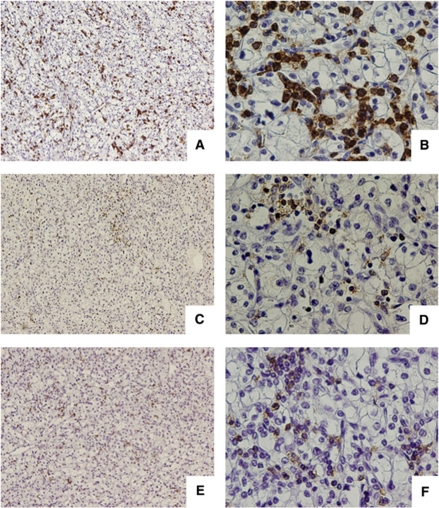
Immunohistochemical staining of tumour-infiltrating lymphocytes in renal cell carcinoma surgical specimen. CD45RO-, CD4-, and CD8-positive cells were observed in the stromal area outside of tumour cells. Representative case of CD45RO (**A** and **B**), CD4 (**C** and **D**), and CD8 (**E** and **F**). Original magnification (**A**, **C**, and **E**) × 100 and (**B**, **D**, and **F**) × 400.

We retrospectively analysed 105 patients with RCC without any preoperative anticancer therapy. By immunohistochemistry, CD45RO+, CD4+, and CD8+ T cells infiltrating into RCC tissue were evaluated. These lymphocytes were detected within cancer nests or present in the stroma in contact to cancer cells (Figure 1). The median number of CD45RO-, CD4-, and CD8-positive cells was 49.8, 16.6, and 18.6, respectively. We defined these median numbers as cutoff values. All cases were classified into low- or high-density groups for each marker, that is, CD45RO+lo, CD4+lo, and CD8+lo (low-density groups) and CD45RO+hi, CD4+hi, and CD8+hi (high-density groups).

### Associations between the density of tumour-infiltrating T cells and clinicopathological features

**Figure 2:**
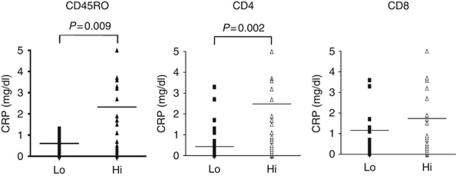
Relationship between preoperative CRP levels and expression of T-cell subset marker (CD45RO, CD4, and CD8). Increased preoperative CRP levels were positively correlated with CD45RO and CD4 status (*P*=0.009 and *P*=0.002, respectively). There was no significant correlation between CRP and CD8 status.

The clinical and pathological characteristics grouped by TIL density with median value cutoffs are summarised in Table 1. The more patients with higher TNM stage and nuclear grade were found in CD45RO+hi and CD4+hi than in CD45RO+lo and CD4+lo. However, this was not observed for CD8. There were no statistically significant associations between all three T-cell markers and patient's age, gender, lymph-node involvement or distant metastases. Preoperative serum CRP levels are known to be prognostic factor and indicate systematic inflammatory response. In this study, 32 patients had positive preoperative CRP and 73 had negative. The positive correlations between preoperative CRP and TNM stage were observed (Table 2). The more patients with higher TNM stage had positive preoperative CRP. Next, we examined the relationship between preoperative CRP levels and T-cell status. As shown in Figure 2, increased preoperative CRP levels were positively correlated with CD45RO and CD4 status, but not with CD8. This indicated that CD45RO+ and CD4+ T cell might have some roles in systematic inflammatory response of RCC patients.

### Clinical outcome

**Figure 3:**
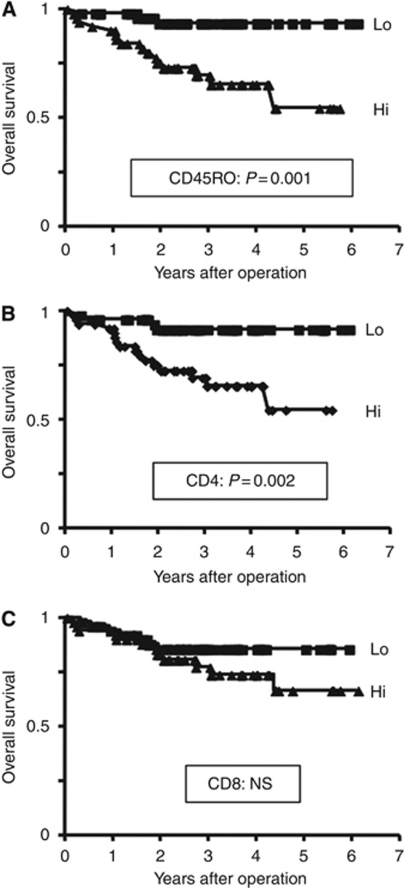
Overall survival of 105 patients with renal cell carcinoma according to tumour-infiltrating T cells. (**A**) CD45RO+hi patients had a significantly poor prognosis than CD45RO+lo patients (*P*=0.001). (**B**) CD4+hi patients had a significantly poor prognosis than CD4+lo patients (*P*=0.002). (**C**) There was no significant difference between CD8+hi and CD8+lo patients (*P*=0.243).

At the time of analysis, 20 of the 105 patients studied had died at median of 15.9 months following surgery (range, 2–52.5). Among the 85 patients who were still alive at last follow-up, the median duration of follow-up was 34.5 months (range, 0.4–73.4). For all subjects estimated overall survival rate 3 and 5 years after surgery was 80.6% and 73.7%, respectively. Univariately, the patients with CD45RO+hi had a significantly worse postoperative prognosis than the CD45RO+lo patients in overall survival rate (hazard ratio, 6.22; 95% confidence interval, 1.88–11.47; *P*=0.001; Figure 3A). In addition, there was also a significant difference between CD4+hi and CD4+lo patients in overall survival rate (hazard ratio, 4.72; 95% confidence interval, 1.64–9.55; *P*=0.002; Figure 3B). By contrast, no significant difference was observed in tumour-infiltrating CD8 T-cell status (hazard ratio, 1.73; 95% confidence interval, 0.69–4.21; *P*=0.243; Figure 3C). In addition, the clinicopathological variables such as T stage, lymph-node involvement, and presence of metastasis, nuclear grade and preoperative CRP levels were all shown to have a significant prognostic impact on overall survival.

A multivariate model was used to identify independent prognostic factors. The model included histopathological variables, T-cell markers, and preoperative CRP levels. This analysis revealed that distant metastases and CD45RO+ status were the markers to show independent prognostic significance (hazard ratio, 9.09 and 5.57; *P*=0.002 and *P*=0.033, respectively; Table 3).

## Discussion

Renal cell carcinoma is considered to be an immunogenic tumour associated with a number of TILs. Previous studies have reported the correlation between TILs and clinical outcome in patients with RCC. In an early small study, increased T-cell infiltration was suggested to be associated with an increased risk for tumour recurrence (Kolbeck *et al*, 1992). More recently, in a larger study, increased mononuclear lymphocyte infiltration in the tumour has been reported to be correlated with poor survival in RCC patients (Webster *et al*, 2006). Nakano *et al* (2001) reported that high densities of CD8+ T cells have worse impact on the prognosis in RCC. Similarly, Igarashi *et al* (2002) also suggested increased CD8+ T cells may be a poor prognostic factor in advanced RCC. However, Bromwich *et al* (2003) reported no significant correlation between CD8+ TIL and overall survival. Our study also indicated no association between CD8+ TIL and survival. Furthermore, several studies have shown more significant impact of CD4+ TIL rather than CD8+ on the prognosis in patients with RCC. Bromwich *et al* (2003) found that increased levels of CD4+ T cells in tumours correlated with poor patient survival that is consistent with our finding. By sharp contrast, the other study reported that increased CD4+ TIL was related with a good prognosis in patients with advanced disease status (Igarashi *et al*, 2002). Taken together, the precise role and differential function of each T-cell subset in RCC tumours remain controversial. In this study, we systematically investigated clinical importance of T-cell subsets infiltrating into human RCC tissues and found that the high density of tumour-infiltrating CD45RO+ as well as CD4+ T cells have negative impact on patient survival. Most importantly, CD45RO status among T-cell subsets was only independent postoperative prognostic marker in RCC as demonstrated by multivariate analysis.

CD45RO is the most suitable single marker for memory T-cell population in human, which could finely represent the activation status of T cell. Therefore, these cells include both CD4+ and CD8+ lymphocytes that have been exposed to antigen. To our best knowledge, none of studies have previously addressed the role of CD45RO+ T cell in RCC. The analysis indicated the positive significant correlations between CD45RO status and advanced pathological features including higher nuclear grade and TNM stage. In addition, besides the presence of distant metastasis, CD45RO status was an independent prognostic marker for RCC patients. These data suggest that CD45RO+ T-cell status has important prognostic value independently of conventional TNM classification in RCC.

C-reactive protein is an indicator of systemic inflammatory response. Previous reports suggested that increased CRP levels predict poor survival in patients with both localised and metastatic RCC (Casamassima *et al*, 2005; Lamb *et al*, 2006; Karakiewicz *et al*, 2007). Furthermore, CRP could be an informative biomarker that reflects disease progression and efficacy of therapeutic intervention. Our data also demonstrated the patients with high CRP level had a significantly poor prognosis than patients with normal CRP level. Furthermore, preoperative CRP levels were positively correlated with CD45RO and CD4 status. This suggests that local inflammatory and immune responses in tumour tissues functionally influence systematic immunological response, thereby leading to clinical outcome in RCC patients. Interestingly, multivariate analysis indicated that CRP status did not reach a significant level for the independent predictors of overall survival in this study. Thus, data further emphasise the importance of CD45RO as a prognostic marker in RCC. However, there are some limitations to draw a definitive conclusion. Approximately 69% of patients evaluated in this study were classified in early stage (stage I or II). Due to this subject imbalance, our results may not have universal validity. Therefore, larger confirmatory studies would be required to validate our data interpretation.

Recent studies have reported that CD45RO+ TILs were associated with better disease outcome for several human cancers. In colorectal cancer, high density of CD45RO+ cells within the tumour was associated with decreased invasiveness, lower stage, and prolonged survival (Pages *et al*, 2005, 2009; Galon *et al*, 2006). Furthermore, in gastric cancer, high numbers of CD45RO+ T cells in tumour tissue were significantly correlated with lower frequencies of lymph-node metastasis or longer survival, and further CD45RO+ TILs were independent prognosis factor (Lee *et al*, 2008). However, this study shows that CD45RO+ T-cell infiltration was associated with poor survival in patients with RCC. This paradoxical phenomenon has been also observed in other T-cell subtypes as previously reported (Nakano *et al*, 2001; Bromwich *et al*, 2003). Although this unique feature seems to be found mostly in RCC but not in other types of cancer, the fundamental reasons are still unknown. There may be complex inhibitory mechanisms including apoptosis, cytokine inhibition, angiogenesis, and other regulatory functions on TILs. Several reports indicate that infiltrating lymphocytes within RCC tumours are often impaired and incapable of mediating tumour rejection. CTL effector function has been reported to be lost following tumour infiltration in murine RCC model (Janicki *et al*, 2008). T cells isolated from TILs of human RCC expressed low granzyme B mRNA levels and did not upregulated its expression upon activation *in vitro* (Kudoh *et al*, 1997). It was also reported that RCC could induce apoptosis in activated tumour-infiltrating T cells by virtue of augmented expression of FasL (Uzzo *et al*, 1999). In addition, the inhibitory interaction of PD-L1/PD-1 has been implicated in RCC (Thompson *et al*, 2007). Furthermore, RCC tumours and TILs produced several cytokines, which inhibit CTL function, including IL-8, TGF-*β*, and IL-10 (Wang *et al*, 1995; Lahn *et al*, 1999). Taken together, RCC tumours possess local mechanisms to inhibit TILs and undermine antitumour immunity. Further studies on memory T cells in correlation with the above potential inhibitory mechanisms are warranted to develop novel therapeutic strategy.

In conclusion, we have found that CD45RO+ memory T-cell status has a significant independent prognostic value, indicating that the adaptive immune response is functionally critical in human RCC.

## Figures and Tables

**Table 1:** Correlation between TIL density and clinicopathological characteristics in the 105 renal cell carcinomas

	**CD45RO**			**CD4**			**CD8**		
	**Lo**	**Hi**	***P***	**Lo**	**Hi**	***P***	**Lo**	**Hi**	***P***
*Age at surgery (years)*									
<60	21	15	0.338	18	18	0.944	18	18	0.944
⩾60	32	37		35	34		35	34	
									
*Gender*									
Female	14	15	0.952	12	17	0.249	15	14	0.874
Male	39	37		41	35		38	38	
									
*T stage*									
pT1	41	28	0.038	40	29	0.116	34	35	0.234
pT2	5	5		5	5		8	2	
pT3	6	18		7	17		10	14	
pT4	1	1		1	1		1	1	
									
*Lymph-node involvement*									
Absent	50	46	0.467	49	47	0.705	50	46	0.282
Present	3	6		4	5		3	6	
									
*Distant metastases*									
Absent	49	40	0.087	49	40	0.087	46	43	0.559
Present	4	12		4	12		7	9	
									
*TNM* *stage*									
I	41	24	0.002	40	25	0.011	34	31	0.379
II	4	3		4	3		5	2	
III	2	13		3	12		5	10	
IV	6	12		6	12		9	9	
									
*Nuclear grade*									
1	29	15	0.011	28	16	0.009	26	18	0.266
2	20	25		22	23		21	24	
3+4	4	12		3	13		6	10	

Abbreviations: TIL=tumour-infiltrating lymphocyte; TNM=tumour-node-metastasis.

**Table 2:** Correlation between CRP and TNM stage

	**CRP**		
	**Negative**	**Positive**	***P***
*TNM stage*			
I	57	8	<0.001
II	6	1	
III	6	9	
IV	4	14	

Abbreviations: CRP=C-reactive protein; TNM=tumour-node-metastasis.

**Table 3:** Multivariate Cox proportional hazards models for the predictors of overall survival

**Variable**	**HR**	**95% CI**	***P***
*CD45RO+ status*			0.033
Lo	1	Referent	
Hi	5.57	1.15–27.12	
			
*CD4+ status*			0.213
Lo	1	Referent	
Hi	2.37	0.61–9.17	
			
*CD8+ status*			0.276
Lo	1	Referent	
Hi	0.28	0.07–1.14	
			
*Primary tumour*			0.213
⩽T2	1	Referent	
>T2	2.1	0.65–6.76	
			
*Lymph-node involvement*			0.384
Negative	1	Referent	
Positive	2.6	0.65–10.52	
			
*Distant metastases*			0.002
Negative	1	Referent	
Positive	9.09	2.24–35.71	
			
*Nuclear grade*			0.254
1	1	Referent	
2	5.52	1.55–20	
3+4	6.94	0.63–71.42	
			
*Preoperative CRP*			0.265
Negative	1	Referent	
Positive	0.45	0.12–1.74	

Abbreviations: HR=hazard ratio; 95% CI=95% confidence interval; CRP=C-reactive protein.
